# Intraspecific and interspecific variations in the synonymous codon usage in mitochondrial genomes of 8 *pleurotus* strains

**DOI:** 10.1186/s12864-024-10374-3

**Published:** 2024-05-10

**Authors:** Wei Gao, Xiaodie Chen, Jing He, Ajia Sha, Yingyong Luo, Wenqi Xiao, Zhuang Xiong, Qiang Li

**Affiliations:** 1 Clinical Medical College & Affiliated Hospital of Chengdu University, Chengdu University, Chengdu, Sichuan China; 2 Key Laboratory of Coarse Cereal Processing, Ministry of Agriculture and Rural Affairs, School of Food and Biological Engineering, Chengdu University, Chengdu, Sichuan China; 3 School of Food and Biological Engineering, Chengdu University, 2025 # Chengluo Avenue, Longquanyi District, Chengdu, Sichuan 610106 China

**Keywords:** Codon usage, Mitochondrial genome, Genetics, Natural selection, Fungi

## Abstract

In this study, we investigated the codon bias of twelve mitochondrial core protein coding genes (PCGs) in eight *Pleurotus* strains, two of which are from the same species. The results revealed that the codons of all *Pleurotus* strains had a preference for ending in A/T. Furthermore, the correlation between codon base compositions and codon adaptation index (CAI), codon bias index (CBI) and frequency of optimal codons (FOP) indices was also detected, implying the influence of base composition on codon bias. The two *P. ostreatus* species were found to have differences in various base bias indicators. The average effective number of codons (ENC) of mitochondrial core PCGs of *Pleurotus* was found to be less than 35, indicating strong codon preference of mitochondrial core PCGs of *Pleurotus*. The neutrality plot analysis and PR2-Bias plot analysis further suggested that natural selection plays an important role in *Pleurotus* codon bias. Additionally, six to ten optimal codons (ΔRSCU > 0.08 and RSCU > 1) were identified in eight *Pleurotus* strains, with UGU and ACU being the most widely used optimal codons in *Pleurotus*. Finally, based on the combined mitochondrial sequence and RSCU value, the genetic relationship between different *Pleurotus* strains was deduced, showing large variations between them. This research has improved our understanding of synonymous codon usage characteristics and evolution of this important fungal group.

**Supplementary Information:**

The online version contains supplementary material available at 10.1186/s12864-024-10374-3.

## Introduction

Codon bias indicates the non-uniform or biased usage of synonymous codons that encode the same amino acid in a gene or genome [1]. The genetic information contained in DNA is transferred to the sequence of 20 amino acids through transcription and translation steps [2]. Among the 64 triplet codon arrangements contained in DNA, 61 triplets can encode 20 standard amino acids, while the other three are translation termination codons. Among the 20 amino acids encoded, 18 amino acids are encoded by multiple different codons, while tryptophan and methionine are encoded by only one codon in most species. The degeneration of the genetic code allows the same amino acid to be encoded by synonymous codons or different codons [3, 4]. However, in most cases, the probability of synonymous codons being used is not random or equal. This common phenomenon is called codon usage bias (CUB) [5–7]. This phenomenon of synonymous codons appearing with different frequencies is often observed in different genes, different organisms, or even the same gene from different species [8–10]. CUB is mainly caused by mutations in the gene coding region, especially mutations in the second or third nucleotides of the codon in the gene coding region [11–13]. A synonymy mutation or “silent mutation” will lead to the variability of synonymous codons in organisms during evolution [14, 15]. Since some codons are more prone to mutation than others, selection can sustain this bias [16]. As a result of GC heterogeneity and GC biased gene transformation (gBGC), codon usage bias may also be a result of local recombination rate-based codon usage bias [17–19]. Consequently, synonymous codons evolve through a combination of mutation, natural selection, and genetic drift of gene translation efficiency, which may play a significant role in genome evolution [20, 21]. There is a mutation mechanism that explains the interspecific differences in codon usage by explaining codon bias by the rate or repair of nucleotide bias or point mutations [22, 23]. Furthermore, the theory of natural selection assumes that synonymous mutations that affect biological adaptability will be favored or suppressed throughout the evolutionary process, leading to changes in the use of codons in genomes or genes [24, 25].

Several cellular processes can be affected by codon bias, including transcription, translation efficiency and accuracy, mRNA stability, protein expression, structure, function, and folding during cotranslation [26–28]. Codon bias affects transcription by altering chromatin structure and mRNA folding, which then affects translation efficiency by affecting translation elongation rate [29, 30]. Therefore, codon bias arises as the result of genome adaptation to transcription and translation mechanisms. The study of molecular evolution of genes benefits from selecting genes that do not change amino acids. The codon bias analysis can reveal evolutionary relationships between closely related organisms because codons are used similarly by closely related organisms [31, 32]. Most highly expressed proteins are encoded by genes with the best codons. With the rapid development of high-throughput sequencing technology, codon bias analysis is now essential for understanding species evolution, environmental adaptation, and genetics, etc [33–35]. In fungal species, particularly in large higher fungi, however, genetic characteristics of codon bias remain unknown [36].

*Pleurotus* is one of the largest cultivated edible fungi in the world, which has rich species diversity [37–40]. Some species of *Pleurotus* are delicious edible fungi, which are widely welcomed by consumers [37, 38, 41]. In addition, *Pleurotus* species also contain a variety of bioactive ingredients, with anti-tumor, antioxidant, anti-inflammatory, anti-virus and other effects [42–45]. Mitochondrial genome, known as the second genome of eukaryotes, plays an important role in maintaining the energy supply of eukaryotic cells [46]. Most fungi have 15 core protein coding genes (PCGs), including *atp6*, *atp8*, *atp9*, *cob*, *cox1*, *cox2*, *cox3*, *nad1*, *nad2*, *nad3*, *nad4*, *nad4L*, *nad5*, *nad6*, and *rps3* [47, 48]. The variation of mitochondrial genome has an important impact on the homeostasis, stress resistance and tolerance, development of eukaryotic cells [49–51]. Our previous research found that the mitochondrial genomes of different *Pleurotus* species had undergone large-scale gene rearrangement, indicating that *Pleurotus* species had undergone significant genetic differentiation [52]. However, the codon bias, genetic characteristics, and evolution of the mitochondrial core PCGs of *Pleurotus* within and between species are still unknown.

In this study, we analyzed and compared the usage characteristics of synonymous codons of mitochondrial core PCGs within and between 8 *Pleurotus* strains, including *P. citrinopileatus*, *P. cornucopiae*, *P. eryngii*, *P. giganteus*, *P. ostreatus* P51, *P. ostreatus*, *P. platypus*, and *P. pulmonarius*. We also deduced the phylogenetic relationship of different *Pleurotus* strains based on relative synonymous codon usage (RSCU) data and compared it with the phylogenetic relationship based on mitochondrial genome sequence inference. This study is the first report to analyze the intraspecific and interspecific synonymous codon usage characteristics of important cultivated edible fungi, which will promote the understanding of the evolution, genetics, and species differentiation of *Pleurotus* species and other related species.

## Materials and methods

### Sequence processing

A total of 8 complete *Pleurotus* mitochondrial genomes have been published in the National Center for Biotechnology Information (NCBI) database, 2 of which were reported by our previous studies [52]. The 8 *Pleurotus* mitochondrial genomes were first downloaded from the NCBI database under the accession numbers NC_036998, NC_038091, NC_033533, NC_062374, OX344747, NC_009905, NC_036999, and NC_061177 [53–57]. We further obtained the core protein coding sequence of the mitochondrial genomes of 8 *Pleurotus* strains. Those core protein coding genes whose sequence length is less than 300 bp were excluded from subsequent analysis [14]. Finally, we obtained 12 core protein coding genes in each *Pleurotus* strains for subsequent analysis, including *atp6*, *cob*, *cox1*, *cox2*, *cox3*, *nad1*, *nad2*, *nad3*, *nad4*, *nad5*, *nad6*, and *rps3*.

### Codon usage indices

The GC3s parameter is used to measure the amount of codons with guanine and cytosine at the third synonymous position, with the exception of Met, Trp, and termination codons [58]. The third base of a codon, which is often the least conserved and most variable position. The codon adaptation index (CAI) is a measure of the bias towards codons that are commonly found in highly expressed genes [59]. CAI reflects the adaptation of a gene’s codon usage to the tRNA pool of the organism, which affects translational efficiency. It is a numerical value ranging from 0 to 1.0, with larger values indicating a greater frequency of synonymous codon usage. The Codon Bias Index (CBI) is a metric for evaluating gene expression, which quantifies the deviation from a random or uniform distribution of codons encoding the same amino acid. The Frequency of Optimal Codons (FOP) is determined by dividing the amount of optimal codons by the total number of synonymous codons in a gene, which provides a direct measure of how often a gene uses the “best” or most efficiently translated codons. The Effective Number of Codons (ENC) is a measure of the number of codons used in a gene, ranging from 20 to 61. A value of 20 indicates that only one codon is used for each amino acid, while 61 indicates that each codon is used on average. A low ENC value (below 35) indicates a strong codon usage preference, while a higher value (above 35) indicates a weak preference. The Relative Synonymous Codon Usage (RSCU) value is calculated by dividing the amino acids encoded by the same codons and their probability of appearing in the same codons, which provides a direct comparison of codon usage across genes or species, accounting for differences in codon composition due to amino acid composition. A value greater than 1 indicates a positive codon bias, while a value less than 1 indicates a negative codon bias. The General Average Hydropathicity (GRAVY) value is determined by summing the hydropathy values of all of the amino acids in the polymerase gene sequences and multiplying them by the number of residues in the gene sequences, which provides insights into the potential membrane-spanning or intracellular localization of a protein. GRAVY values range from − 2 to 2, with positive and negative values representing hydrophobic and hydrophilic proteins, respectively. The Aromaticity (AROMO) value is an indicator of the frequency of aromatic amino acids (Phe, Tyr, and Trp). Aromatic amino acids have a unique chemical structure that confers stability and specific interactions with other molecules. The aromaticity of a protein can affect its structure, function, and interactions with other molecules. GRAVY and AROMO values are also indicators of amino acid usage, and changes in amino acid composition will also affect the results of codon usage analysis. All of these codon usage indicators can be calculated using CodonW1.4.2 [60] or CAIcal server [61].

### Neutrality plot analysis

The neutrality plot (GC12 vs. GC3) can be used to analyze the balance between mutation and selection when codon bias is formed. GC12 represents the average GC content in the first and second positions of the codon (GC1 and GC2), while GC3 represents the GC content in the third position. Neutral evolution theory assumes that mutations occur randomly and have no effect on the fitness of the organism. However, selection pressure can introduce biases in the observed mutation frequencies, leading to deviations from neutrality [62]. A strong statistical correlation between GC12 and GC3 indicates that the species is mainly driven by mutation, whereas a lack of correlation implies the main driving force is natural selection.

### ENC-GC3s plot analysis

The ENC-GC3s plot (ENC vs. GC3s) is typically employed to assess whether the codon usage of a particular gene is impacted solely by mutation or other factors, such as natural selection. This diagram consists of the ordinate ENC value and abscissa GC3s value, with an expected curve calculated via a specific formula [63]. If the corresponding points are distributed around the expected curve, mutation pressure is an independent force in the formation of codon bias. However, if the points are significantly lower or distant from the expected curve, some other factors, such as natural selection, likely play a key role in the formation of codon bias.

$${\rm{ENC}}{\;_{{\rm{exp}}}}\; = \;2\; + \;{\rm{GC}}{3{\rm{s}}}\; + \;{{29} \over {{\rm{GC}}_{\rm{s}}^2\; + \;{{{\rm{(}}1\; - \;{\rm{G}}{{\rm{C}}{\rm{s}}}{\rm{)}}}^2}}}$$

The ENC_Ratio_ value reflects the variation range between the expected value and the actual value of ENC.

$${\rm{ENC}}{\;_{{\rm{Ratio}}}}\; = \;\;{{{\rm{EN}}{{\rm{C}}_{{\rm{exp}}}}\; - \;{\rm{EN}}{{\rm{C}}_{{\rm{obs}}}}} \over {{\rm{EN}}{{\rm{C}}_{{\rm{exp}}}}}}$$

### PR2-Bias plot analysis

Additionally, the Parity Rule 2 bias (PR2-Bias) plot analysis based on [A3/(A3 + U3) vs. G3/(G3 + C3)] can be utilized to determine the degree and direction of the gene bias. The center point in the plot is A = T and C = G, meaning the codon has no usage bias.

### Correspondence analysis

Correspondence analysis (COA) is a widely accepted multivariate statistical analysis method used to identify codon usage patterns. All genes were placed in a 59-dimensional hyperspace, taking into account the 59 sense codons (Met and Trp excluded). This method can detect the main trends in codon usage in the core CDS of *Pleurotus* and arrange codons along the axis according to the RSCU value.

### Determination of optimal codons

The genes were ordered from highest to lowest expression according to the ENC value, and 10% of the genes from the front and rear ends were selected to form a high- and low-expression gene dataset. The D-value between the RSCU of the two datasets (ΔRSCU) was then calculated, with ΔRSCU values greater than 0.08 being defined as codons with high expression. Codons with RSCU values greater than 1 were considered high-frequency codons. A codon with ΔRSCU > 0.08 and RSCU > 1 was defined as the optimal codon.

### Phylogenetic analysis

The phylogenetic relationships of *Pleurotus* strains were compared between codon usage-based and mitochondrial sequence-based methods. Using the RSCU values of the 8 *Pleurotus* strains, SPSS v19.0 software was employed to generate a hierarchical clustering method to illustrate the relationship tree between the different species. We employed the method described in our previous studies [48, 64] to construct phylogenetic trees of the 8 *Pleurotus* strains using the combined mitochondrial gene datasets. To do this, individual mitochondrial genes were aligned using MAFFT v7.037 [65], and then the aligned sequences were combined into a single set using Sequence Matrix v1.7.8 [66]. Potential phylogenetic conflicts between different mitochondrial genes were identified through a partition homogeneity test. Partition Finder 2.1.1 [67] was used to determine the most suitable model of partitioning and evolution for the combined mitochondrial gene set. The phylogenetic tree was constructed using the Bayesian inference (BI) method with MrBayes v3.2.6 [68]. Two independent runs with four chains (three heated and one cold) were conducted for 2 × 10^6^ generations, with samples taken every 100 generations. The first 25% of samples were discarded as burn-in, and the remaining trees were used to calculate Bayesian posterior probabilities (BPP) in a 50% majority-rule consensus tree. *Ganoderma lingzhi* was set as the outgroup [69, 70].

## Results

### Nucleotide composition of *Pleurotus* core PCGs

The codon usage analysis of 12 mitochondrial core PCGs from 8 *Pleurotus* strains revealed that the average length of these genes ranged from 370 bp to 2262 bp, with the *nad3* gene having the shortest average length and the *rps3* gene having the longest. Out of these 12 core PCGs, 10 genes had varying sequence lengths among the different *Pleurotus* species, while the *cox2* and *nad6* genes had the same gene length across all 8 strains. The *rps3* gene showed the greatest length variation, with a maximum difference of 318 bp. Different *Pleurotus* species show great differences in base composition, even between the same species (*P. ostreatus*). The base composition of these 12 core PCGs was found to be rich in T base, with an average content of 41.80%, followed by A base, with an average content of 31.70%. The G and C base contents were relatively low, with an average of 13.59% and 12.92%, respectively. The average GC content of core PCGs ranged from 19.36 to 33.57%, with the *rps3* gene having the lowest GC content and the *cox1* gene having the highest.

### Codon usage analysis

**Fig. 1:**
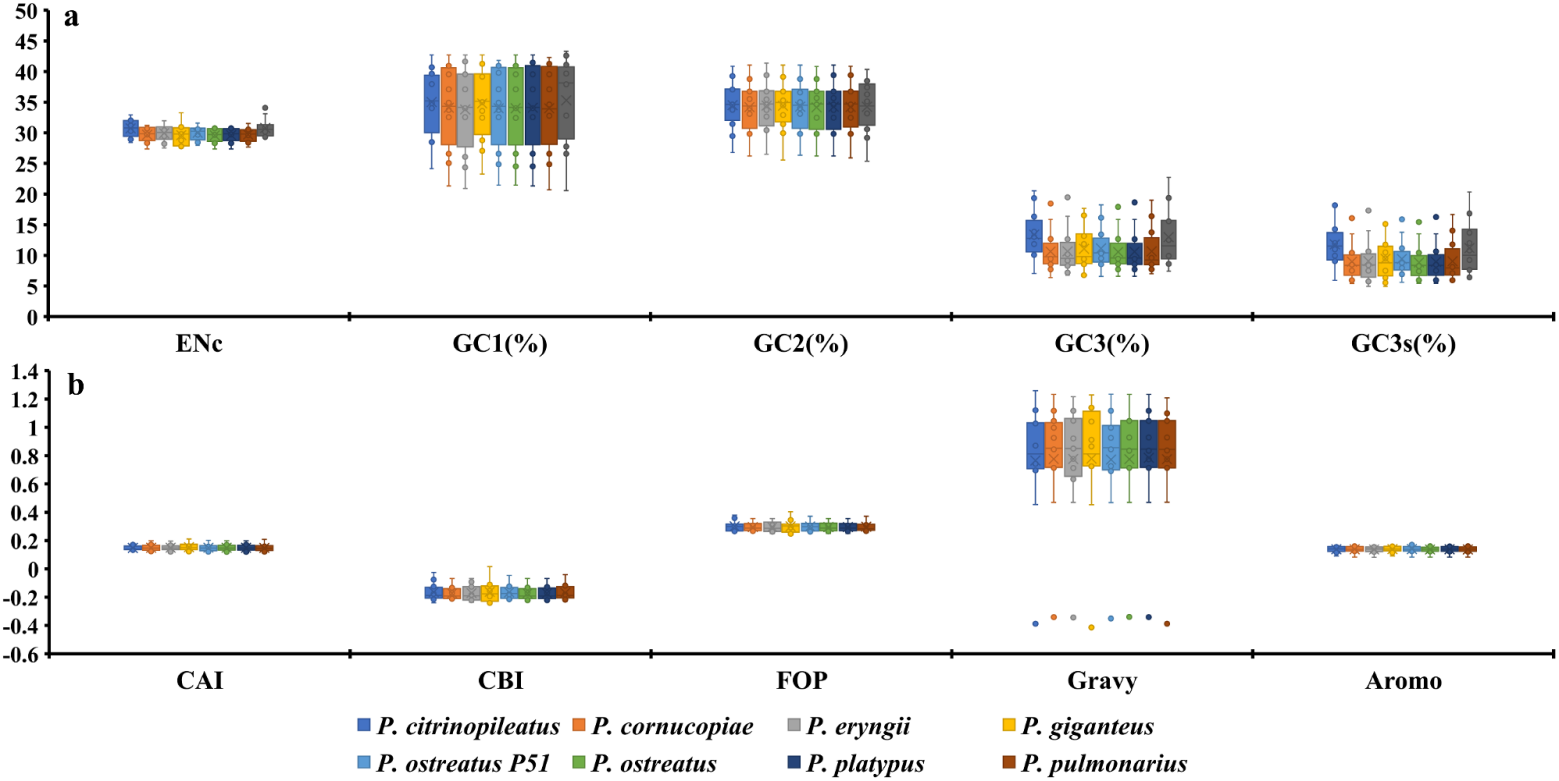
Codon usage indicators of 12 mitochondrial core protein coding genes in different *Pleurotus* strains

The GC1, GC2 and GC3 contents of the 12 core PCGs in the 8 *Pleurotus* strains were 34.23%, 34.31% and 11.08%, respectively (Fig. 1). The average GC3s value of these 12 PCGs was 9.44%, indicating that the mitochondrial core PCGs of *Pleurotus* tend to end with an A or T base. Additionally, the indices of A3s, T3s, G3s, and C3s of the 12 core PCGs of *Pleurotus* species showed that the codons were more likely to end with A, followed by T, C and G, with values of 54.80%, 54.65%, 7.53%, and 2.27%, respectively. We conducted an analysis of the codon bias of 12 core PCGs in 8 *Pleurotus* strains. The CAI values of the core PCGs ranged from 0.12 to 0.20, with *nad2* having the lowest value and *nad3* having the highest. *P. giganteus* had the highest CAI value, while *P. citrinopileatus* and *P. pulmonarius* had the lowest, indicating that they had a strong codon bias. The CBI values of the 8 *Pleurotus* strains ranged from − 0.164 to -0.173, with *P. giganteus* having the lowest value and *P. ostreatus* having the highest. The average FOP values of the 12 core PCGs ranged from 0.25 to 0.37, with *nad1* having the lowest value and *nad3* having the highest. *P. ostreatus* and *P. platypus* had the lowest FOP value, while *P. giganteus* had the largest. The GRAVY values of the 12 core PCGs were mostly positive, indicating that they were likely hydrophobic proteins, with the exception of *rps3*, which was considered hydrophilic. The AROMO values of the PCGs ranged from 0.08 to 0.17, with *rps3* having the highest value and *cox3* having the lowest. The AROMO values of the 8 *Pleurotus* strains were relatively similar, with an average of 0.14. The two *P. ostreatus* species showed differences in various base bias indicators, among which *P. ostreatus* P51 had high CBI, FOP, ENC, GC3s and Aromo values, while *P. ostreatus* had higher CAI and Gravy indicators, indicating that the frequency of base synonymous codon usage also changed within *Pleurotus* species.

### Codon usage correlation analysis

**Fig. 2:**
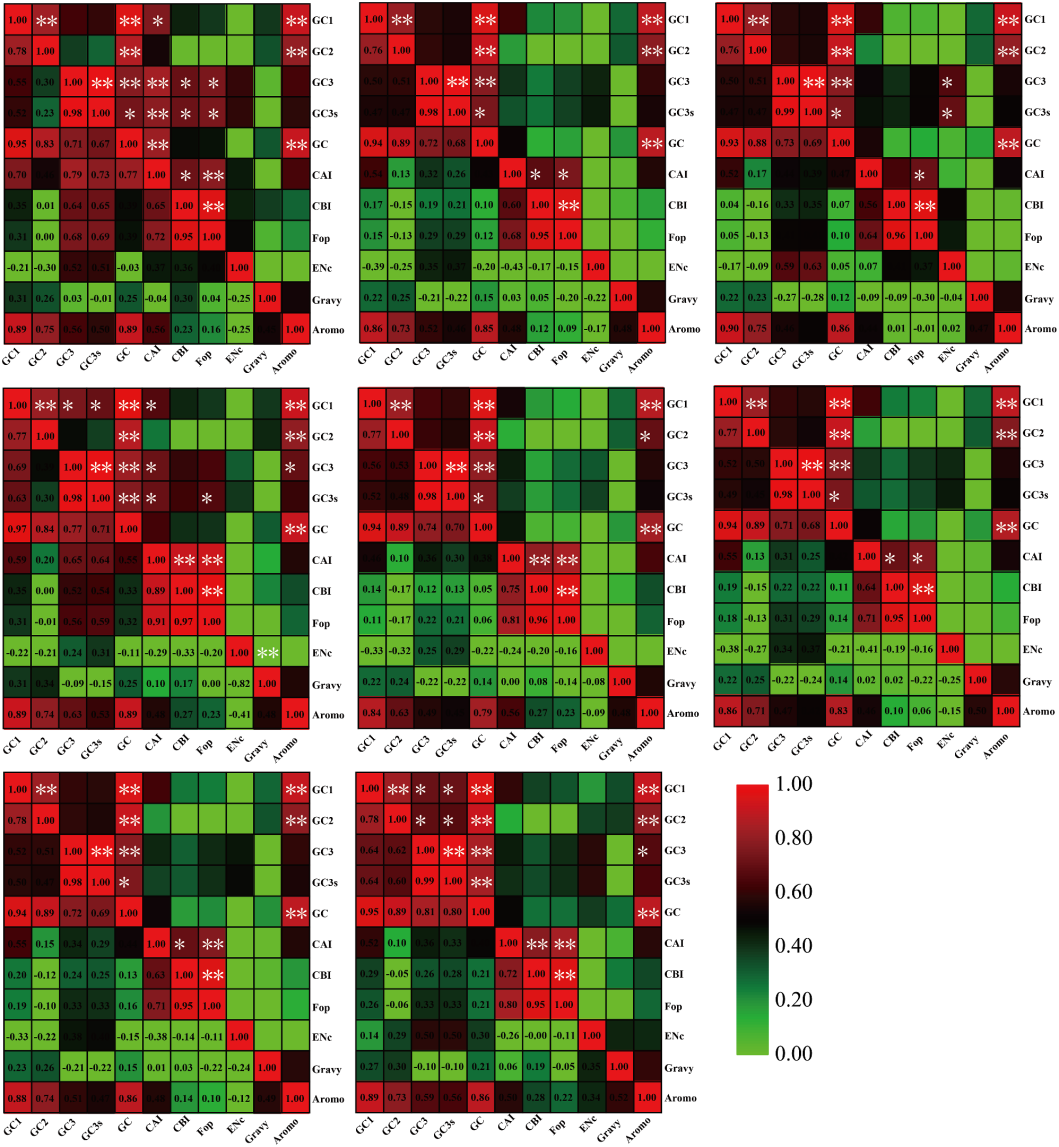
Pearson’s correlation analysis heatmap of different codon usage indicators of 8 *Pleurotus* strains. The color of the color block changes from green to red, indicating that the correlation index is increasing. One asterisk indicates a significant correlation between the two indicators at the *P* < 0.05 level, while two asterisks indicate a significant correlation between the two indicators at the *P* < 0.01 level. The 8 *Pleurotus* species are *P. citrinopileatus*, *P. cornucopiae*, *P. eryngii*, *P. giganteus*, *P. ostreatus* P51, *P. ostreatus*, *P. platypus*, and *P. pulmonarius*, from left to right and from top to bottom

A significant correlation was observed between the GC1 content of mitochondrial codons and GC2, GC3, GC3s, and AROMO values in all eight *Pleurotus* strains (*P* < 0.05) (Fig. 2). Furthermore, a significant correlation was found between the GC2 content and GC content and AROMO values (*P* < 0.05). GC3 content was significantly correlated with GC3s and GC content (*P* < 0.05), and it was also found to affect codon bias in two *Pleurotus* species (*P. citrinopileatus*, and *P. giganteus*). GC3s and GC content were significantly correlated in all eight *Pleurotus* strains (*P* < 0.05). Additionally, the GC content was found to be significantly correlated with the AROMO values in all *Pleurotus* strains (*P* < 0.01). Furthermore, the CAI index of mitochondrial codons was significantly correlated with the FOP index and CBI index in seven out of eight *Pleurotus* strains (*P* < 0.05). Lastly, a negative correlation was observed between the ENC value and GRAVY value in *P. giganteus* (*P* < 0.01).

### Neutrality plot analysis

**Fig. 3:**
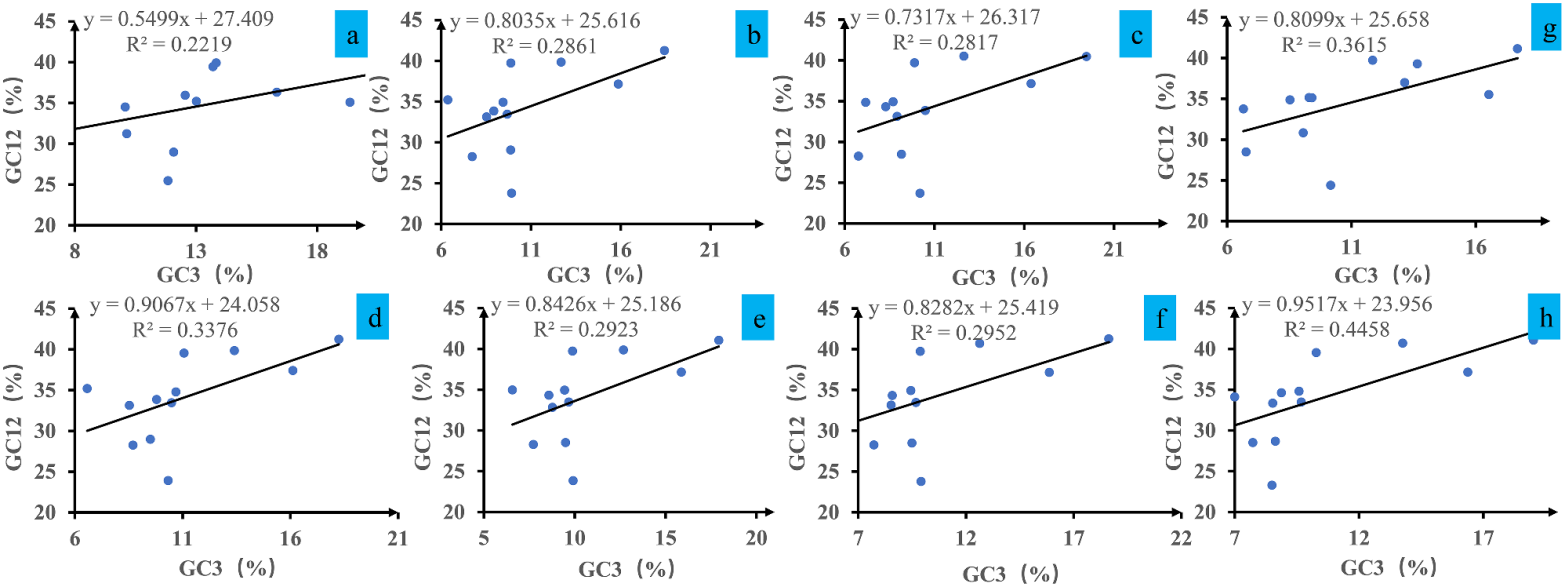
Neutrality plot analysis of GC12 and the third codon position (GC3) for the entire coding DNA sequence of 8 *Pleurotus* strains. a, *P. citrinopileatus*; b, *P. cornucopiae*; c, *P. eryngii*; d, *P. giganteus*; e, *P. ostreatus* P51; f, *P. ostreatus*; g, *P. platypus*; h, *P. pulmonarius*

We calculated the relationships between GC12 and GC3 based on neutrality plot analysis (Fig. 3). The GC12 content varied from 23.29 to 41.25%, and the GC3 content varied from 6.37 to 20.53%. The analysis between GC12 and GC3 content in mitochondrial codons of *Pleurotus* revealed a weak positive correlation, with the regression coefficient ranging from 0.55 to 0.95 and the R^2^ value ranging from 0.2219 to 0.4458. Statistical analysis showed that there was no significant correlation between GC12 and GC3 values (*P* > 0.05), indicating that natural selection played a major role in codon bias of *Pleurotus*.

### ENC-GC3s plot analysis

**Fig. 4:**
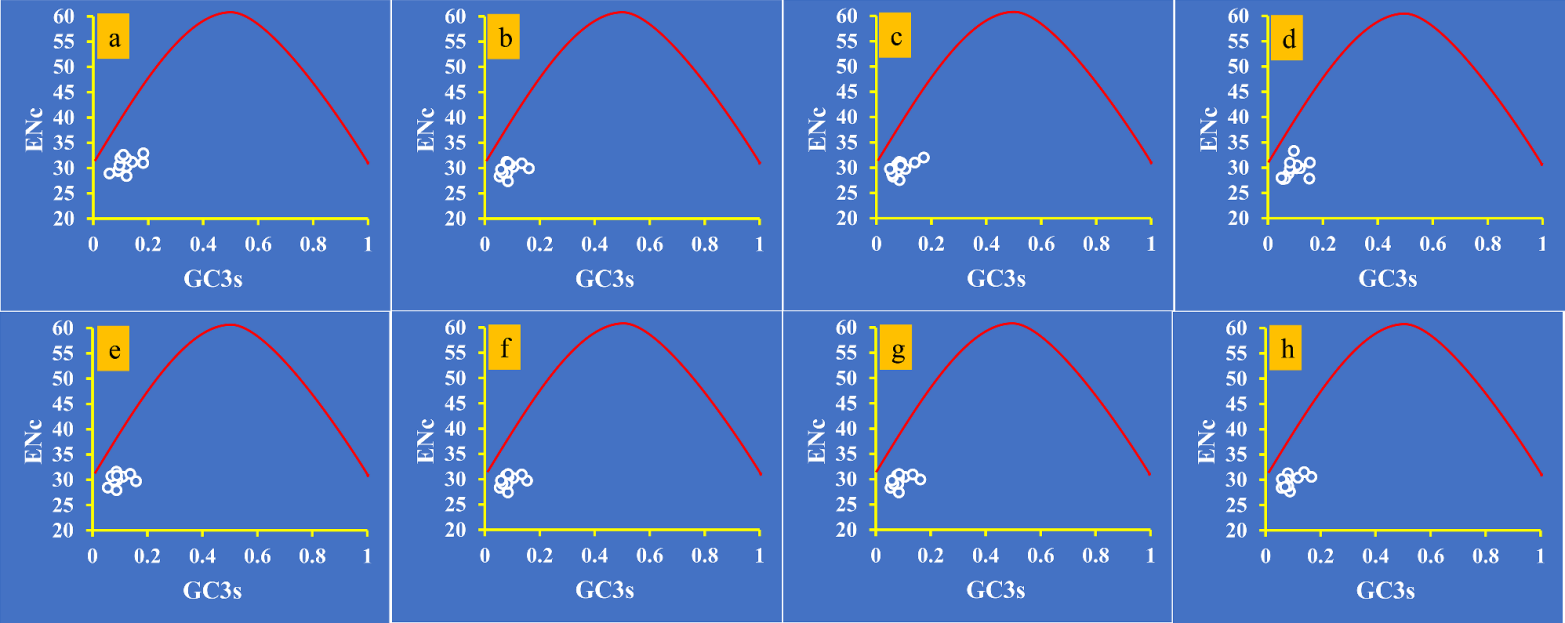
ENC-GC3 plot analysis of 12 core PCGs in 8 *Pleurotus* strains. The solid line represents the expected curve when codon usage bias is affected only by mutation pressure. a, *P. citrinopileatus*; b, *P. cornucopiae*; c, *P. eryngii*; d, *P. giganteus*; e, *P. ostreatus* P51; f, *P. ostreatus*; g, *P. platypus*; h, *P. pulmonarius*

The average ENC value of all 12 core PCGs detected was found to be 29.86, which is lower than 35, indicating a strong codon usage preference (Fig. 1). Moreover, the ENC values of 8 *Pleurotus* strains ranged from 29.58 to 30.74, further confirming the strong codon usage preference of *Pleurotus* species. The ENC plot showed that all *Pleurotus* genes were below the expected ENC-plot curve (Fig. 4), indicating that factors other than mutation pressure, such as natural selection, play a role in codon bias formation.

Additionally, the ENC_Ratio_ values for all core PCGs ranged from 18.59 to 20.55%, indicating that the expected values were greater than the actual values (Fig. S1). This demonstrates that GC3s have an important influence on the formation of codon bias. In conclusion, it can be inferred that natural selection is a major factor determining the formation of *Pleurotus* codon bias.

### PR2-Bias plot analysis

**Fig. 5:**
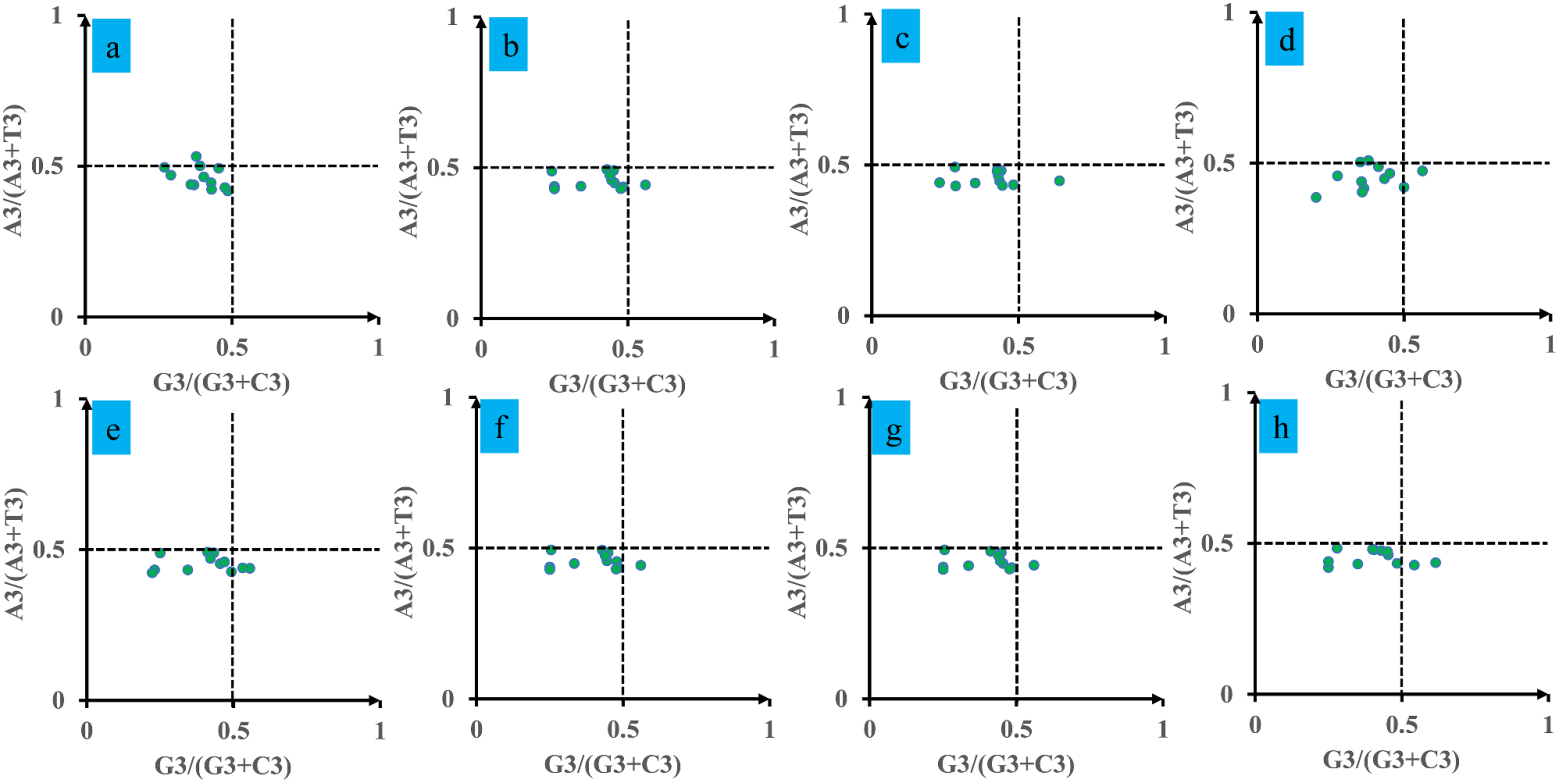
Parity Rule 2 (PR2) plot analysis of 12 core PCGs in 8 *Pleurotus* strains. a, *P. citrinopileatus*; b, *P. cornucopiae*; c, *P. eryngii*; d, *P. giganteus*; e, *P. ostreatus* P51; f, *P. ostreatus*; g, *P. platypus*; h, *P. pulmonarius*

We conducted a Parity Rule 2 (PR2) plot analysis to investigate whether *Pleurotus* mitochondrial genes have any biases (Fig. 5). Both axes were centered on 0.5 to divide the plot into four quadrants. The results showed that the third base of the mitochondrial codon of *Pleurotus* had a strong preference for T over A and C over G. Most of the dots were found to be distributed in the third quadrant, while six out of the eight strains were not distributed in the fourth quadrant (preferring A to T and C to G), with *P. citrinopileatus* and *P. giganteus* being the exception. All the 8 *Pleurotus* strains were not distributed in the first quadrant (preferring A to T and G to C). This suggests that strong codon usage preference exist in *Pleurotus* species.

### Correspondence analysis

**Fig. 6:**
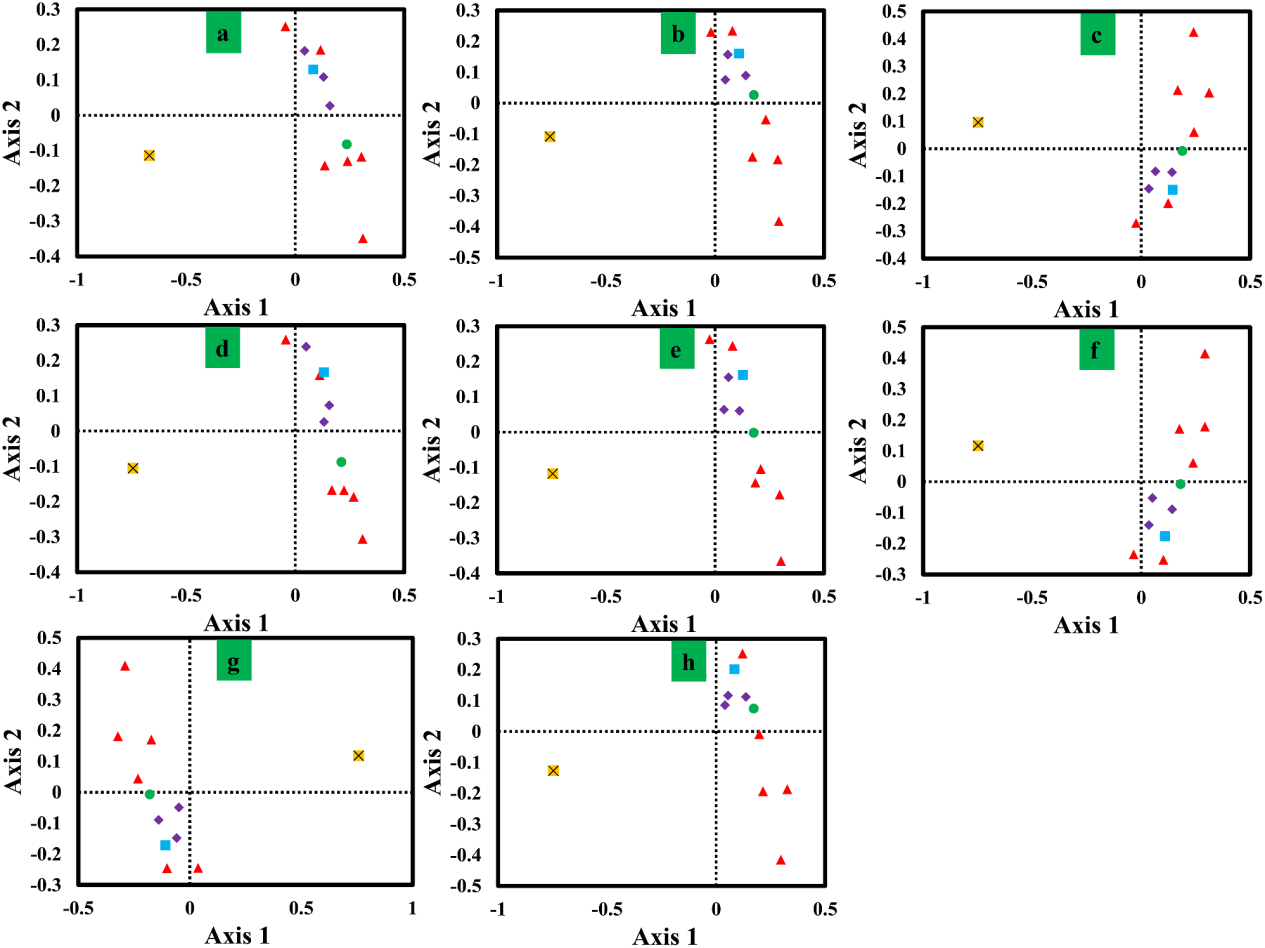
Correspondence analysis (COA) based on the relative synonymous codon usage (RSCU) values of 12 mitochondrial genes from 8 *Pleurotus* strains. Purple represents the *cox* gene, red represents the *nad* gene, green represents the *atp6* gene, blue represents the *cob* gene, and yellow represents the *rps3* gene. a, *P. citrinopileatus*; b, *P. cornucopiae*; c, *P. eryngii*; d, *P. giganteus*; e, *P. ostreatus* P51; f, *P. ostreatus*; g, *P. platypus*; h, *P. pulmonarius*

To further analyze codon biases in *Pleurotus*, we conducted a correspondence analysis (COA) based on the RSCU values of mitochondrial genes from the 8 *Pleurotus* strains (Fig. 6). Axis 1, Axis 2, Axis 3 and Axis 4 are the main contributors to variance, with average contribution rates of 45.61%, 15.62%, 8.24% and 6.30%, respectively. The results showed that Axis 1 was the largest contributor to variance. Pearson correlation analysis showed that Axis 1 had significant correlation with CAI and ENC values. Additionally, we observed large variation in the *rps3* gene and other core PCGs, indicating the differentiation of synonymous codon usage of core PCGs.

### Optimal codon analysis

**Fig. 7:**
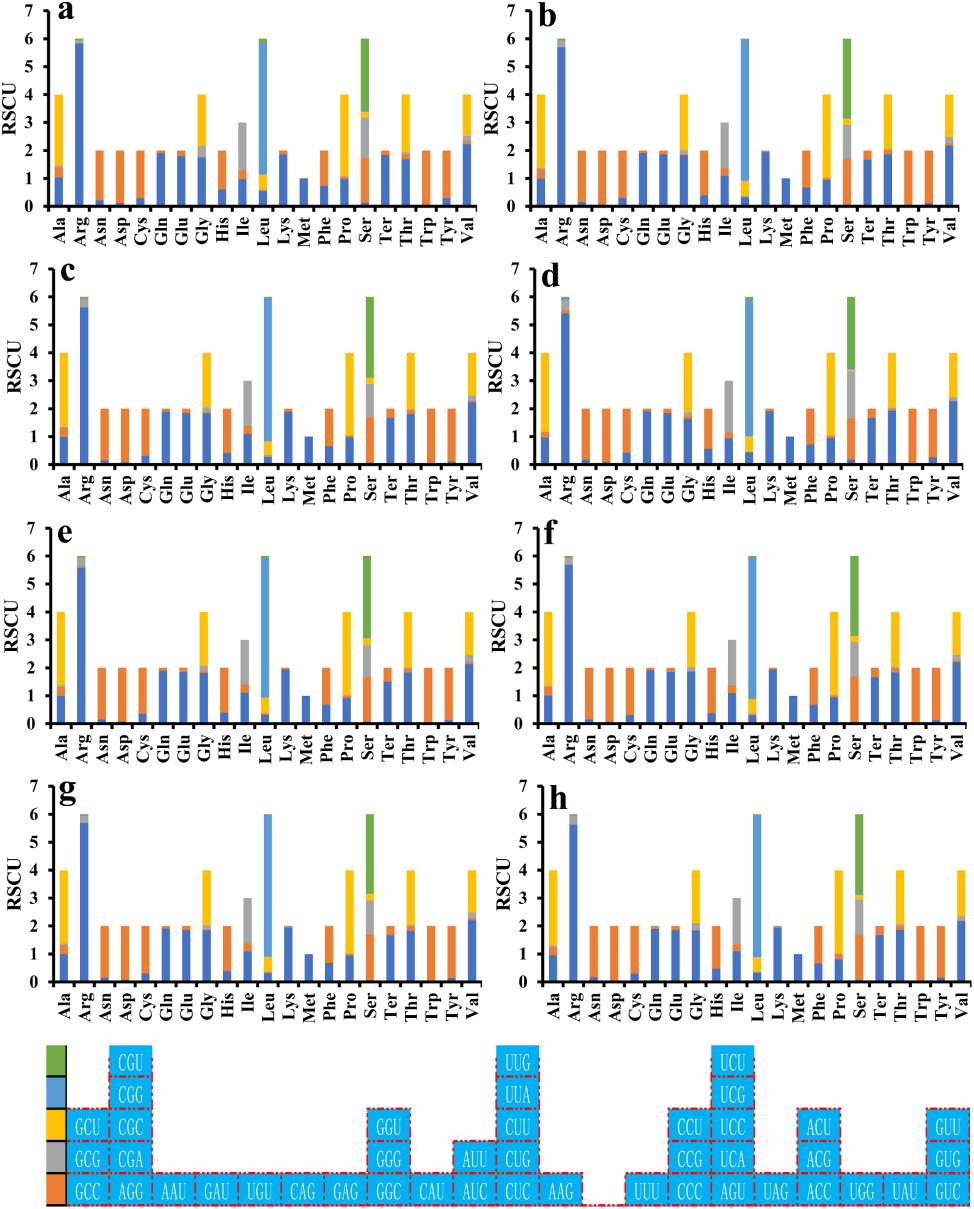
Relative synonymous codon usage (RSCU) analysis of 12 mitochondrial genes from 8 *Pleurotus* strains. The color blocks with different colors on the bottom vertical axis represent different codons in the image above. a, *P. citrinopileatus*; b, *P. cornucopiae*; c, *P. eryngii*; d, *P. giganteus*; e, *P. ostreatus* P51; f, *P. ostreatus*; g, *P. platypus*; h, *P. pulmonarius*

**Fig. 8:**
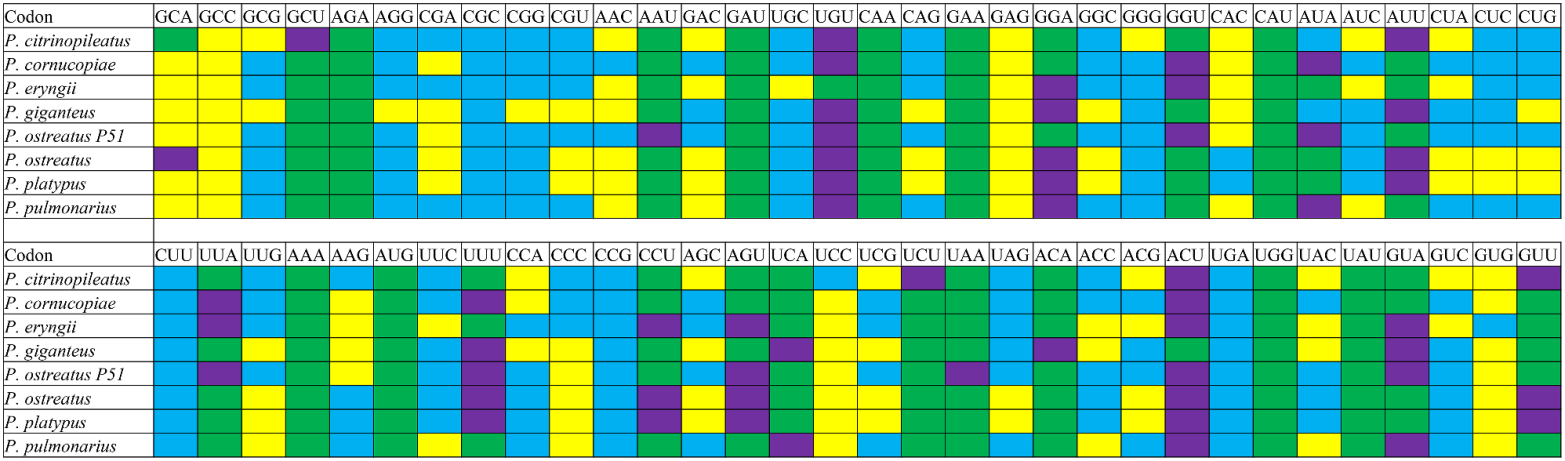
Optimal codons of 8 *Pleurotus* strains (ΔRSCU > 0.08 and RSCU > 1), which are marked in purple. Highly expressed codons (ΔRSCU > 0.08) were marked in yellow and high-frequency codons (RSCU > 1) were marked in green

Analysis of the Relative Synonymous Codon Usage (RSCU) of eight *Pleurotus* strains revealed 27 high-frequency codons in six species (*P. citrinopileatus*, *P. cornucopiae*, *P. eryngii*, *P. ostreatus* P51, *P. platypus* and *P. pulmonarius*), with *P. giganteus* containing 26 and *P. ostreatus* containing 28 (Fig. 7). AUA was found to be used at a low frequency in *P. citrinopileatus* and *P. giganteus*, but used at a high frequency in other *Pleurotus* species. Of the 28 frequently used codons, 15 ended in T, 11 in A, and only 2 in G, indicating a preference for codons ending in A/T. In addition, 22, 15, 23, 30, 19, 28, 28, and 20 highly expressed codons (ΔRSCU > 0.08) were identified in the 8 *Pleurotus* strains, including *P. citrinopileatus*, *P. cornucopiae*, *P. eryngii*, *P. giganteus*, *P. ostreatus* P51, *P. ostreatus P. platypus*, and *P. pulmonarius*, respectively (Fig. 8). Comparative analysis revealed that 6, 6, 7, 7, 10, 9, 8, and 6 optimal codons (ΔRSCU > 0.08 and RSCU > 1) were found in *P. citrinopileatus*, *P. cornucopiae*, *P. eryngii*, *P. giganteus*, *P. ostreatus* P51, *P. ostreatus P. platypus*, and *P. pulmonarius*, respectively. All of these optimal codons ended with A/T, with UGU and ACU being the most widely used, followed by GGA, AUU, and UUU, which were used as the optimal codons of five strains. GCA, GCU, AAU, UCU, UAA, and ACA were each used as the optimal codons of one species. Furthermore, *P. ostreatus* P51 and *P. ostreatus* showed great differences in the use of optimal codons. AAU, GGU, AUA, UUA, UAA, and GUA were used as the optimal codons in *P. ostreatus* P51, while GCA, GGA, AUU, CCU, and GUU were used as the optimal codons in *P. ostreatus*.

### Phylogenetic analysis

**Fig. 9:**
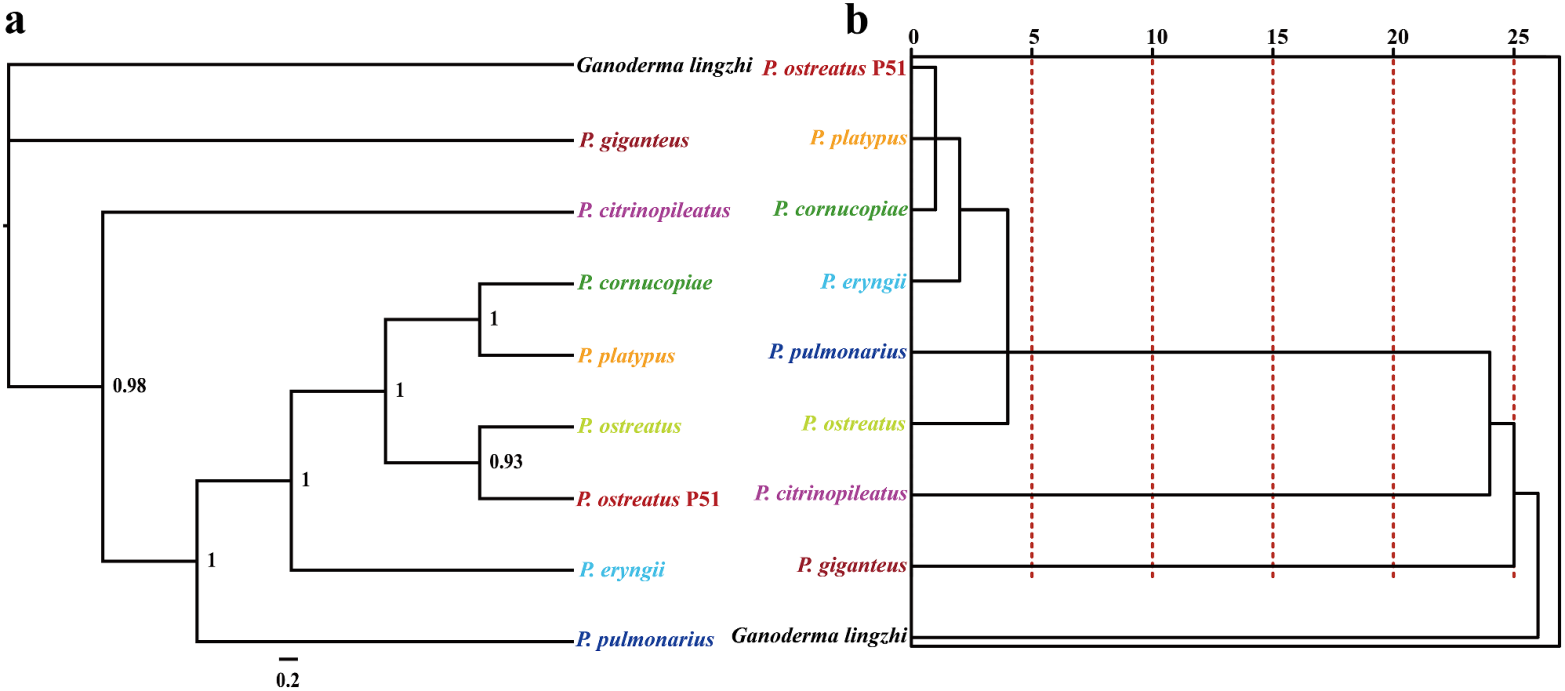
Relationship inference of different *Pleurotus* strains based on the Bayesian inference (BI) (**a**) method and relative synonymous codon usage (RSCU) hierarchical clustering (**b**)

The Bayesian inference (BI) method was employed to construct phylogenetic trees of 8 *Pleurotus* strains based on the combined mitochondrial gene set (Fig. 9). The results demonstrated that *P. giganteus* and *P. citrinopileatus* had diverged from the *Pleurotus* population earlier. *P. cornucopiae* was identified as the sister species of *P. platypus*. Furthermore, two *P. ostreatus* strains were grouped in the same evolutionary clade, which indicated their close phylogenetic relationship. In contrast to the phylogenetic relationship inferred from sequences, the species relationship inferred from RSCU had some discrepancies, such as the phylogenetic status of *P. ostreatus*, *P. eryngii*, and *P. pulmonarius*. Nevertheless, the RSCU-based species relationship also clearly revealed the close relationship between *P. platypus* and *P. cornucopiae*, as well as the early divergence of *P. giganteus* and *P. citrinopileatus* from the *Pleurotus* population.

## Discussion

The development of sequencing technology has enabled researchers to gain access to the genetic sequences of various species and types of genomes, including the nuclear genome, chloroplast genome and mitochondrial genome [71–73]. Through the analysis of genetic information, it has been observed that the usage of synonymous codons varies among different species, with some codons being used more frequently than others [74]. This codon usage bias is mainly affected by several factors, such as gene base composition, gene length, gene expression level, tRNA abundance, amino acid hydrophobicity, aromaticity, mutation, and selection, with mutation and selection being the most influential [75, 76]. Examining the codon bias characteristics of different species can help to understand the genetic structure and evolution trend of species [77, 78]. However, the codon usage of important organelle genomes of higher fungi has not been thoroughly studied.

The mitochondrial genome is often referred to as the ‘second genome’ of eukaryotes. In this study, it was found that the length and base composition of mitochondrial core PCGs of different *Pleurotus* strains varied significantly, even within the same *Pleurotus* species, indicating the differentiation of *Pleurotus* mitochondrial genes. The differences in synonymous codons were mainly reflected in the third codon. Additionally, it was observed that all core PCGs of *Pleurotus* species tend to end with A/T, which is in line with the rule of mitochondrial codon usage in many eukaryotes [79, 80]. The majority of high-frequency codons parsed by RSCU also end with A/T, further confirming the tendency of using the third codon of *Pleurotus*. Moreover, variations in base usage were observed among different species and genes. The two *P. ostreatus* species also showed differences in various base bias indicators, including CAI, CBI, FOP, ENC, and GC3s values, indicating that the frequency of base synonymous codon usage also changed in the within *Pleurotus* species. Furthermore, correlations were detected between codon base composition and GC3s, CAI, CBI, and FOP, suggesting the influence of base composition on codon bias. An ENC value lower than 35 indicates a strong codon preference [81, 82]. The average ENC value of the mitochondrial core PCGs of *Pleurotus* was found to be 29.86, which indicates strong codon preference. Furthermore, the expected and actual ENC values showed significant differences (18.59-20.55%). Neutrality plot analysis and PR2-Bias plot analysis also showed evidence of natural selection in *Pleurotus* codon bias. This is consistent with the results seen in the mitochondrial genomes of other species [83–85]. The findings of this study revealed that, despite some discrepancies in codon usage indicators between different *Pleurotus* species, they all experienced strong natural selection on their mitochondrial PCGs.

Mitochondria are believed to have been obtained from bacteria by the ancestors of eukaryotes [86], and most mitochondrial genes have since been transferred to the nuclear genome [87]. While most eukaryotes still retain some core PCGs, some tRNA genes and rRNA genes for energy metabolism [88, 89], which can be used as a molecular marker for phylogeny. As such, the mitochondrial genome is considered a useful tool for inferring phylogenetic relationships of species [90–92]. In this study, the genetic relationship of different *Pleurotus* species was analyzed based on a combined mitochondrial gene set and high support rates were found for each evolutionary clade. Additionally, the relationship between different *Pleurotus* species was determined based on their RSCU values, which differed from the sequence-based relationships. The phylogenetic tree constructed with RSCU values can serve as a supplement and reference for constructing mitochondrial gene phylogenetic trees, which agreed with previous research [93, 94]. Codon bias, the non-uniform usage of synonymous codons, plays a role in species biodiversity, physiology, morphology, and nutrition of fungi. It can contribute to species-specific genetic signatures, influence translational efficiency and protein expression levels, potentially affect protein structure and function related to morphology, and influence the ability of a species to utilize different nutrients. However, the precise mechanisms and causal relationships between codon bias and these biological characteristics remain incompletely understood [95, 96]. Consequently, this research enhanced the comprehension of codon usage characteristics and genetic evolution of this higher fungal group.

### Electronic supplementary material

Below is the link to the electronic supplementary material.

Supplementary Material 1

## Data Availability

All data generated or analyzed during this study are included in this published article [and its supplementary information files].
