# Supplementary material for: Intraspecific and interspecific variations in the synonymous codon usage in mitochondrial genomes of 8 pleurotus strains
Source: BMC Genomics. 2024 May 10;25:456. doi: 10.1186/s12864-024-10374-3 (PMC11084086; doi:10.1186/s12864-024-10374-3)
Supplement: Supplementary file 1 — Supplementary Material 1 [file 12864_2024_10374_MOESM1_ESM.docx]

**Intraspecific and interspecific variations in the synonymous codon usage in mitochondrial genomes of 8 *Pleurotus* strains**

**Running title: Codon bias of 8 *Pleurotus***

**Wei Gao^a^, Xiaodie Chen^b^, Jing He^b^, Ajia Sha^b^, Yingyong Luo^b^, Wenqi Xiao^b^, Zhuang Xiong^b^, Qiang Li*^b^,**

**a Clinical Medical College & Affiliated Hospital of Chengdu University, Chengdu University, Chengdu, Sichuan, China;**

**b Key Laboratory of Coarse Cereal Processing, Ministry of Agriculture and Rural Affairs, School of Food and Biological Engineering, Chengdu University, Chengdu, Sichuan, China.**

***Corresponding author:**

**Qiang Li (**[**leeq110@126.com**](mailto:leeq110@126.com)**)**

**Phone: +86-028-84616653;**

***Present address: School of Food and Biological Engineering, Chengdu University, 2025 # Chengluo Avenue, Longquanyi District, Chengdu** **610106, Sichuan, China.**

**
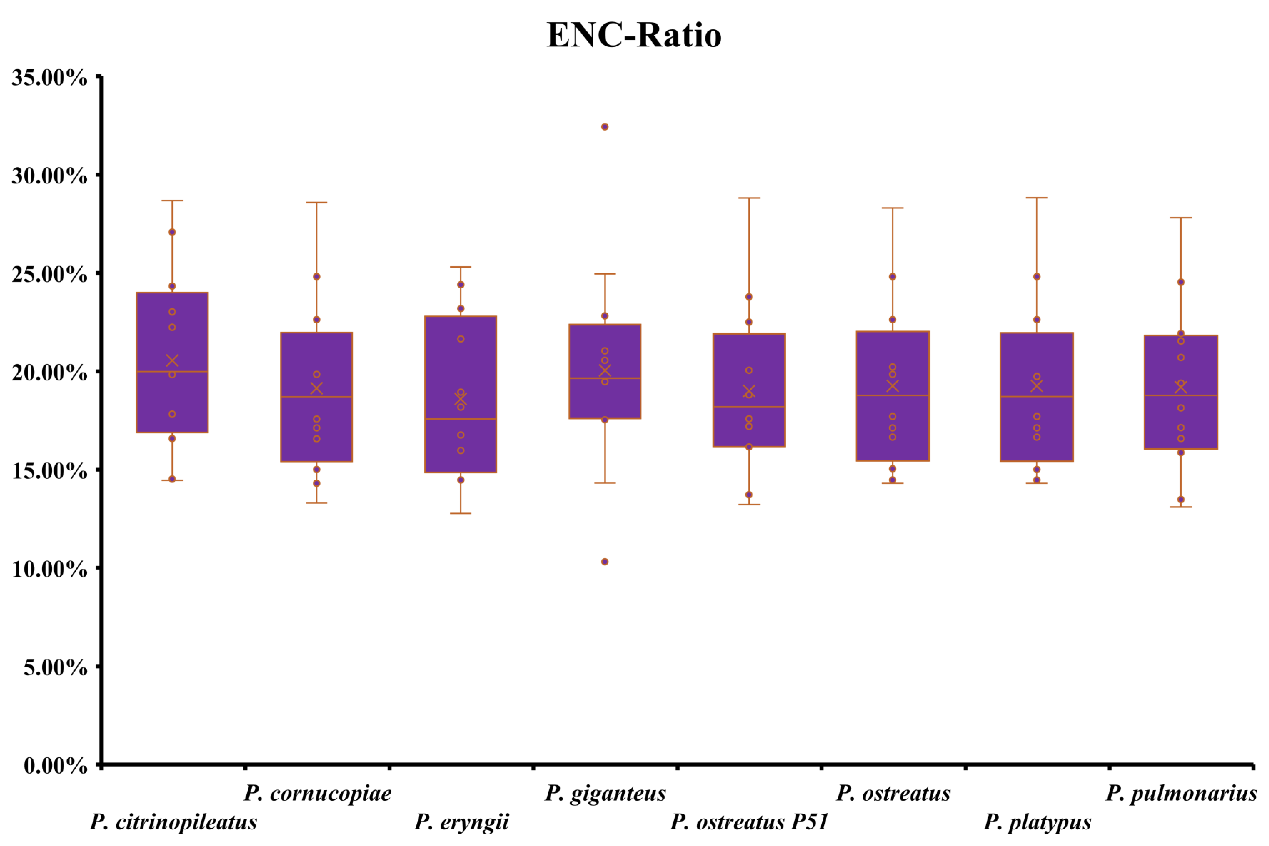
**

**Supplementary Figure S1** Variability of expected and actual ENC values of 12 mitochondrial genes from 8 *Pleurotus* strains.
